# Models of Integration for Mental Health and HIV/AIDS Among Adolescents and Young People in Low- and Middle-Income Countries: A Scoping Review

**DOI:** 10.3390/ijerph23050589

**Published:** 2026-04-30

**Authors:** Puleng Lydia Ramphalla, Mantji Juliah Modula, Mutshidzi Mulondo

**Affiliations:** 1 Division of Public Health, Faculty of Health Sciences, University of Free State, Bloemfontein 9300, South Africa; mulondoma@ufs.ac.za; 2 School of Nursing, Faculty of Health Sciences, University of Free State, Bloemfontein 9300, South Africa; modulamj@ufs.ac.za

**Keywords:** adolescents, young people, HIV, mental health, service integration

## Abstract

**Highlights:**

**Public health relevance—How does this work relate to a public health issue?**

- Adolescents and young people (AYP) in low- and middle-income countries experience a double burden of HIV and various mental disorders, which interact to worsen health outcomes, treatment adherence, and long-term well-being.
- This scoping review maps and synthesizes current HIV-mental health integration models for AYP in resource-limited settings to fill the gap in literature in this important public health area.

**Public health significance—Why is this work of significance to public health?**

- Findings demonstrate that integrated mental health–HIV service delivery is feasible and acceptable, even in resource-limited settings when approaches are contextualized and participatory.
- By identifying common barriers and facilitators, the review generates implementation-relevant evidence needed to inform scalable and sustainable adolescent health programming.

**Public health implications—What are the key implications or messages for practitioners, policy makers and/or researchers in public health?**

- Practitioners: mental health integration in routine HIV services is feasible but require adequate training, continuous supervision, and clear referral pathways.
- Policy makers: Strengthening adolescent HIV outcomes will require policy-level commitment to integrated mental health–HIV programs, including workforce development, inclusive data systems and sustainable financing beyond pilot projects. Researchers: There is a critical need for rigorous and longitudinal studies that assess effectiveness, sustainability, and clinical outcomes of integrated mental health–HIV models for adolescents across diverse LMIC contexts.

**Abstract:**

Adolescents and young people (AYP) experience a disproportionate burden of both mental health conditions and HIV, particularly in low- and middle-income countries (LMICs)-nations classified by the World Bank as having lower or middle economies. Mental health problems such as depression, anxiety, and substance use increase HIV (Human Immunodeficiency Virus that attacks the human immune system and leads to various illnesses when untreated) risk, and negatively affect treatment adherence and outcomes. However, mental health remains insufficiently integrated into HIV research and programming. Evidence on how mental health services are operationally integrated into HIV prevention and treatment for this population is limited and fragmented. This scoping review mapped existing evidence on the integration of mental health services into HIV treatment programs for AYP in LMICs, guided by PRISMA-ScR (a guideline used for reporting scoping reviews in research) and the Person–Concept–Context framework, a framework used to define specific research question in research. In this case, the population was adolescents and young people (10–24 years) receiving HIV prevention or treatment services, the concept referring to the integration of mental health interventions such as screening, assessment and counseling within HIV services, and the context focused on low- and middle-income countries (LMICs). PubMed, MEDLINE, Scopus and PsycINFO databases were searched for studies published between 2014 and 2024. Eligible studies reported mental health screening, assessment, treatment, or referral within HIV services for AYP in LMICs. Two reviewers independently screened studies, assessed full texts, and extracted data. Of 634 records identified, ten (10) studies met the inclusion criteria. All were conducted in Sub-Saharan Africa and primarily used qualitative or pilot designs. Four integration approaches were identified: routine mental health screening within HIV services, task-shifting to trained lay providers, peer-led and community-based psychosocial support, and culturally adapted, youth-centered psychological interventions. Common barriers included stigma, low mental health literacy, limited training and supervision, staffing constraints, and weak referral systems. Existing evidence is limited, remains exploratory, preliminary, and largely focused on feasibility and implementation experiences, suggesting that integrating mental health services within adolescent HIV care in LMICs may be feasible and acceptable when approaches are contextually adapted and participatory.

## 1. Introduction

The World Health Organization (WHO) is a specialized technical assistance United Nations (UN) authority responsible for leading and coordinating global public health issues. Mental health is a fundamental component of the World Health Organization’s definition of health as a state in which an individual is free from any physical, mental and social illnesses or disorders, and is able to adequately handle daily life challenges as an active member of society [1].

Globally, more than one in ten adolescents are affected by a mental disorder, and about 40% of these are anxiety or depressive disorders [2]. Despite being globally recognized as an important public health issue, mental health is still less prioritized as a disease burden in many low-and middle-income countries (LMICs); yet more than 70% of the global mental health burden occurs in these countries [3].

According to the United Nations Development Agenda, Sustainable Development Goal (SDG) 3 aims to ensure healthy lives and promote well-being for all at all ages [4]. SDGs are global goals adopted by countries to improve the lives of their citizens by 2030. The agenda includes targets to end communicable diseases like HIV/AIDS, preventing and treating non-communicable diseases and mental health, and strengthening health systems to achieve universal health coverage. This particular SDG 3 acknowledges that good health is crucial for individuals to lead full, productive lives and for societies to flourish. Therefore, addressing HIV, mental health, and related substance abuse is not only a more effective and sustainable approach and health priority, but also a powerful driver for achieving SDG 3, creating healthier individuals, stronger communities, and more resilient health systems. Most African countries bear the bigger burden of HIV/AIDS and have competing health and development priorities with insufficient funds to adequately address mental health conditions. As a result, mental healthcare is severely underfunded. Data recorded in health management systems do not include mental health, which contributes to an underappreciation of the disease burden in countries across the continent [5]. Lack of data means that policymakers cannot comprehend the depth of the problem that countries are facing.

The relationship between mental health and HIV is complex and bidirectional. Poor mental health serves as a risk factor for HIV infection by influencing high-risk behaviors, worsening disease progression, and reducing adherence to treatment. Conversely, living with HIV often leads to or intensifies mental health conditions such as depression, anxiety, trauma, and substance use disorders [6,7,8,9].

Adolescence represents a crucial period of growth and development, marked by significant psychological, social, and biological transitions [5]. The age category ‘youth ‘or ‘young person’ tends to be fluid across countries and socio-cultural and economic contexts. Many countries use the terms ‘young person’ and ‘adolescent’ interchangeably depending on the context. The United Nations defines ‘youth’ as anyone between the ages of 15 and 24 [10,11]. The World Health Organization also defines ‘Adolescents’ as persons aged 10–19 years, ‘Youth’ as the age range 15–24, and ‘Young People’ as the age range 10–24 years [12]. This means there are definition overlaps on the age range of 15–19-old people who can be classified both as adolescents and young people; however, these definitions do not override what countries may ultimately choose to adopt as working definitions for their contexts. For the purpose of this review, adolescents and young people (AYP) refer to individuals aged 10–24 years, a population whose health outcomes are vital to long-term community well-being.

During adolescence, individuals are especially vulnerable to mental health problems and to behaviors that increase the risk of HIV infection. Globally, the prevalence of mental health disorders among adolescents continues to rise. There is evidence that 34.6% of mental health disorders begin as early as 14 years of age, and up to 62.5% by the age of 25 [13]. According to the WHO’s Mental Health of Adolescents Factsheet [14], mental disorders account for approximately 13% of the global disease burden among adolescents aged 10–19 years. Suicide remains the fourth leading cause of death among young people aged 15–29 years [14,15]. Adolescents living with HIV generally have a higher prevalence of mental health conditions (e.g., depression and anxiety) compared with their HIV-negative peers [16]. Treating comorbid mental illnesses such as depression, anxiety, and substance use disorders can improve adherence to care and clinical outcomes for people who have been diagnosed with or living with HIV (PLHIV). These figures underscore the urgent need for focused interventions addressing both mental health and HIV among young people.

Untreated mental health problems, particularly depression and substance use are linked to increased HIV risk behaviors, resulting in higher morbidity and mortality related to HIV [17]. Despite growing evidence of these interconnections, mental health needs among people living with or at risk of HIV remain largely unmet, particularly in LMICs. In most health systems, mental health services are typically accessed only when symptoms become severe, or a formal diagnosis has been made [18].

Existing studies on integrating mental health and HIV services vary widely by setting, population, and delivery models. In addition, prior research and reviews have largely focused on general populations [19,20,21,22] or the burden of mental health conditions among people living with HIV [23,24,25], with limited attention to how these mental health services are integrated into HIV services.

This gap is particularly pronounced for adolescents and young people in low- and middle-income countries, where evidence remains sparse and fragmented. Much of the literature focuses on prevalence or adult-focused interventions, offering little insight into integration models, implementation strategies, and the contextual factors shaping delivery for this population.

A scoping review is therefore warranted to systematically map existing evidence on mental health–HIV service integration for adolescents and young people, identify implementation approaches, and synthesize key barriers, facilitators, and knowledge gaps.

### Aim and Objectives

The purpose of this scoping review was to map and synthesize existing evidence on the integration of mental health services into HIV prevention and treatment programs for adolescents and young people in LMICs.

This is the first review to:

1. Identify models of integration between mental health and HIV services implemented for adolescents and young people in LMICs;
2. Examine how these integration models influenced outcomes in this target population;
3. Identify the facilitators and barriers reported in implementing different integration approaches for mental health and HIV.

## 2. Materials and Methods

### 2.1. Registration of the Scoping Review Protocol

The scoping review was registered with Open Science Framework (OSF), a free and open-source platform developed and maintained by Center for Open Science, Washington, DC, USA. The registration DOI is https://doi.org/10.17605/OSF.IO/VB2J7.

### 2.2. The Study Design

The review was conducted in line with the Joanna Briggs Institute (JBI) methodology for scoping reviews and is reported according to the PRISMA Extension for Scoping Reviews (PRISMA-ScR, 2018) [26,27], and the completed PRISMA-ScR checklist is available in the Supplementary Materials (Table S1). The review followed the recommended JBI steps, including formulation of the review questions using the Population–Concept–Context framework, development and implementation of a systematic search strategy, independent screening and selection of studies, data extraction using a predefined charting tool, and synthesis and presentation of findings in a transparent and reproducible manner.

The eligibility criteria and review questions were developed using the PCC where the population (Person) comprised adolescents and young people receiving HIV prevention or treatment services; the concept focused on the integration of mental health interventions, including screening, assessment, treatment, or referral, within HIV care; and the context was limited to low- and middle-income countries (LMICs).

### 2.3. The Database Search

In addition to a librarian-assisted search, a comprehensive literature search was conducted across four scientific databases, (PsycINFO, MEDLINE, Scopus and PubMed) to identify relevant studies. The search strategy combined both index terms and free-text keywords to capture the breadth of evidence on the integration of mental health into HIV prevention and treatment services for adolescents and young people in low- and middle-income countries (LMICs). The search terms were formulated based on the Person–Concept–Context (PCC) framework to ensure alignment with the population (adolescents and young people), the concept (integration of mental health services), and the context (LMIC settings). The search was limited to studies published in the English language between 2014 and 2024. Reference lists and citations of all primary articles retrieved during the initial search were also screened to identify additional relevant studies not captured in the database searches and searched through Google Scholar. The following key search terms and index words were used individually and in different combinations with Boolean operators (AND/OR): “HIV”, AND “mental health”; “Youth” OR “Adolescent mental health”; “HIV AND mental health integration”; “Integration models”; and “Adolescent HIV AND mental health”

The final search string was:

(“HIV” OR “human immunodeficiency virus”);

AND (“mental health” OR depression OR anxiety OR “psychological intervention”);

AND (“adolescent” OR “youth” OR “young people”);

AND (“integration” OR “service integration” OR screening OR “task-shifting” OR; “psychosocial”) AND (“developing countries” OR “low- and middle-income country” OR LMIC).

Gray literature was not included in the search; priority was given to peer-reviewed studies to ensure comparability across studies and allow for a structured, reproducible search strategy within time and resource constraints of the review.

### 2.4. Inclusion of Data Sources

This review covered a ten-year period of studies published between 2014 and 2024, and was limited to English-language publications to capture recent evidence on the integration of mental health services within HIV programs for adolescents and young people in low- and middle-income countries (LMICs). Eligible studies reported mental health screening, assessment, counseling, treatment, or referral integrated within HIV prevention or treatment services for adolescents and young people.

Study selection followed the PRISMA-ScR screening process. All identified records were imported into Rayyan systematic review software for screening and management. Two reviewers independently screened titles and abstracts against the predefined inclusion and exclusion criteria. Studies that did not meet the eligibility criteria were excluded. Full-text articles were subsequently assessed using the same criteria to determine final eligibility. Any discrepancies in records selection were resolved through discussion and consensus between the reviewers.

### 2.5. Data Extraction

A standardized data charting form was developed by the principal investigator to systematically extract relevant information from each included study. The charting form captured key study characteristics including citation, objectives, methodology, study population, country or setting, type of integration model, and any reported outcomes.

The database search yielded 634 records, including PubMed (*n* = 512), PsycINFO (*n* = 56), MEDLINE (*n* = 26), and Scopus (*n* = 40). Prior to screening, a total of 153 duplicative records were removed, and 481 records remained for screening. During this stage, 437 records were excluded because they did not meet the inclusion criteria. The main reasons for exclusion included studies conducted in different settings (*n* = 33), targeting populations other than adolescents and young people (*n* = 159), ineligible publication types such as reviews and protocols (*n* = 62), or studies addressing different research objectives (183) such as prevalence of mental health among people living with HIV.

Following the screening stage, 41 articles were assessed for eligibility. Of these, 31 articles were excluded after full-text review due to similar ineligibility reasons above.

Ultimately, 10 studies met the inclusion criteria and were included in the final synthesis.

Since aim of this scoping review was to map the breadth and nature of the available evidence rather than to evaluate study quality, a formal assessment of methodological quality or risk of bias was not undertaken. This is consistent with JBI guidance and the PRISMA-ScR framework for scoping reviews. 

**Figure 1:**
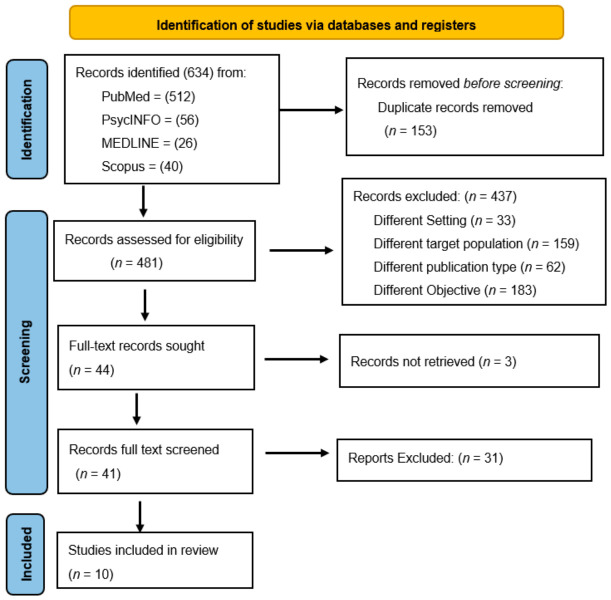
PRISMA flow diagram showing screening and final inclusion of studies.

The Study selection process and inclusion of studies is summarized in the PRISMA flow diagram in Figure 1 below [26].

### 2.6. Data Analysis

The studies were analyzed using a descriptive and narrative approach to make sense of the evidence across the included studies. We first summarized key study characteristics, including setting, design, population, and type of mental health–HIV integration approach. We then examined themes across studies to understand how integration was implemented, what worked, and what challenges were commonly reported. Given the diversity of study designs and outcomes, findings were synthesized qualitatively, with attention to both implementation insights and potential limitations.

## 3. Results

### 3.1. Description of the Studies

This section provides a summary description of the included studies. The review included ten English-language studies between 2014 and 2024. The studies were conducted in LMICs, specifically in the Sub-Saharan Africa.

The geographic distribution included South Africa (*n* = 3), Kenya (*n* = 2), Uganda (*n* = 2), Tanzania (*n* = 1), and Malawi (*n* = 1), with one study conducted across four countries (Malawi, Tanzania, Uganda, and Zambia).

A range of study designs was represented. Qualitative studies (*n* = 3) and mixed-methods studies (*n* = 3) were the most common. In addition, pilot or quasi-experimental studies (*n* = 2) evaluated intervention implementation approaches, while one study used a prospective cohort design (*n* = 1), and one study used a randomized controlled trial (*n* = 1).

Most studies (*n* = 6) evaluated early-stage pilot integration models, with emphasis on mental health screening and psychosocial interventions, while four studies (*n* = 4) examined routine integrated service delivery within HIV programs. Across the included studies, reported outcomes primarily related to feasibility and acceptability (*n* = 6), while four studies (*n* = 4) reported outcomes related to service uptake, screening, or detection of mental health conditions among adolescents and young people receiving HIV services.

Table 1 below summarizes the characteristics of included studies; highlighting geographic coverage, study design, target population as well as integration approach reported outcomes in each study.

### 3.2. Integration Models Across Studies

The reviewed studies adopted different study methods to demonstrate diverse but complementary models of integrating mental health services into HIV care for adolescents and young people (AYP) in low- and middle-income countries. Across the ten included studies, four broad integration models were identified: (1) structural or system-level integration, (2) task-shifting and capacity-building approaches, (3) peer-led and community-based models, and (4) culturally adapted psychological interventions. These models illustrate different strategies through which mental health interventions have been incorporated into HIV services for AYP.

#### 3.2.1. Structural or System-Level Integration

Two [23,28] studies examined structural integration of mental health screening within routine HIV clinical services. One study [23] conducted in South Africa evaluated routine screening for mental health and substance use among adolescents receiving antiretroviral therapy- (a combination of medications used to treat HIV) using validated screening tools including PHQ-9 (a itemizes questionnaire tool used to measure severity of depression), GAD-7 (screening tool used to severity of anxiety in people), PC-PTSD-5 (a brief screening tool used to identify possible post-traumatic stress disorder-(PTSD), and CAGE-AID (a brief screening questionnaire used to detect possible substance or drug abuse). The study involved a large cohort of more than 1000 adolescents aged 10–19 years and reported high screening coverage (over 90%), suggesting that routine mental health screening could be implemented within adolescent HIV services. However, although referral pathways were available, the study did not clearly document follow-up procedures after referral, which limited the understanding of subsequent treatment outcomes.

The second study examined the integration of the HEADSS psychosocial screening tool within adolescent “teen club” HIV programs in Malawi [28]. This qualitative study explored providers’ perspectives on implementing routine psychosocial screening within HIV services. Key challenges identified included limited provider capacity, stigma related to mental health and HIV, lack of culturally adapted screening tools, and weak referral systems. Although the tool had previously been validated in other contexts, it had not been culturally adapted for Malawi, which may have influenced its implementation.

#### 3.2.2. Task-Shifting and Capacity-Building Approaches

Several studies [29,30,31] explored task-shifting strategies that involved training non-specialist providers to deliver mental health interventions within HIV programs.

One study evaluated the acceptability of group interpersonal psychotherapy (IPT-G) delivered by community health workers to postpartum adolescents living with HIV in Kenya [29]. The intervention was reported to improve interpersonal relationships, reduce depressive symptoms, and strengthen social support networks. However, the study was conducted in a single clinic and had a short follow-up period, limiting conclusions about long-term implementation.

A qualitative study conducted in Kenya [31] explored stakeholder perspectives on integrating psychological interventions for depression within HIV care and treatment centers. The study involved interviews with adolescents, caregivers, and healthcare providers and identified several barriers to integration, including low mental health literacy, stigma, inadequate training, unclear referral pathways, and staff shortages.

Similarly, Petersen et al. [31] explored the perceptions of young South African women on ART who received motivational interviewing and problem-solving therapy delivered by lay health workers. Participants reported improved coping strategies and greater understanding of the relationship between alcohol use, mental health, and ART adherence.

#### 3.2.3. Peer-Led and Community-Based Integration

Two studies [32,33] explored peer-led or community-based psychosocial support models integrated within HIV programs.

Laurenzi et al. [32] conducted a mixed-methods study across Malawi, Tanzania, Uganda, and Zambia to adapt a peer-support intervention for young mothers living with HIV. The study used a participatory co-development approach to adapt the psychosocial component of the Ask-Boost-Connect-Discuss (ABCD) peer-support model. The intervention emphasized peer engagement and community support networks and was reported to be acceptable to participants. However, the study focused specifically on young mothers living with HIV, which may limit generalizability to other adolescent populations.

Harrison et al. [33] evaluated the “Better Together” peer-support group program implemented in South Africa for adolescents and young adults living with chronic conditions, including HIV. The program involved facilitated group sessions designed to promote peer interaction and psychosocial support. Participants reported improved resilience, reduced internalized stigma, and stronger social support networks through engagement with peers experiencing similar health challenges.

#### 3.2.4. Culturally Adapted Psychological Interventions

Three [34,35,36] studies examined psychological interventions that were culturally adapted for adolescents living with HIV in LMIC settings.

Khamisi et al. [34] conducted a mixed-methods study in Uganda to adapt a mindfulness and acceptance-based intervention known as the Discoverer-Noticer-Advisor–Values (DNA-V) model for adolescents receiving ART. This intervention is intended to help AYP develop psychological flexibility through awareness, exploration and values-guided actions. The adaptation process involved tailoring intervention language, metaphors, and materials to ensure cultural relevance and feasibility. Although the intervention demonstrated high acceptability, the small sample size limited conclusions regarding effectiveness.

Musanje et al. [35] later evaluated a group-based mindfulness and acceptance intervention derived from the DNA-V model using an open-label randomized trial among adolescents aged 15–19 years receiving ART in Uganda. The intervention improved psychological flexibility but did not significantly improve self-reported ART adherence.

In addition, Njau et al. [36] explored the integration of psychological interventions for depression within HIV clinics in Tanzania, highlighting contextual factors influencing implementation such as stigma, limited mental health literacy, and shortages of trained providers.

#### 3.2.5. Summary

Across the included studies, integration approaches ranged from structural screening models embedded in HIV clinics to task-sharing, peer-support, and culturally adapted psychological interventions. Most studies focused on feasibility, acceptability, and early implementation experiences rather than long-term clinical outcomes.

## 4. Discussion

The studies reviewed collectively highlight diverse but convergent approaches to integrating mental health services within HIV care for adolescents and young people in resource-limited settings with recurring themes, facilitators and barriers. Table 2 synthesizes key implementation insights across existing models for integrating mental health services into HIV care for AYP in LMICs. The findings demonstrate that although integration approaches vary widely across settings, four dominant models consistently emerge: structural integration of mental health screening within HIV services, task-shifting to non-specialist providers, peer-led community support models, and culturally adapted psychological interventions.

Although structural integration models demonstrated success in identifying common mental health conditions, key considerations for this approach include structured and continuous capacity building and supervision for health care providers. Facilities must also be adequately staffed, as integrated screening can increase workload, particularly when screening tools are lengthy. Addressing structural barriers such as private space for screening, patient flow, staff workload, leadership support, and engagement remains key to the successful structural integration model [28]. Some of these barriers, especially patient factors, are supported by Rutakumwa et al. [37], where longer patient waiting times undermined the benefits of structural integration in under-staffed settings. High screening coverage alone and increased identification of mental health conditions without further treatment is neither useful nor ethical. Unclear referral pathways in these models not only undermine the impact of the screening but also raise ethical concerns regarding failure to provide treatment where indicted.

According to the Joint United Nations Programme on HIV/AIDS(UNAIDS), a UN agency leading and coordinating global HIV/AIDS response, screening must be accompanied by access to diagnostic assessment and relevant follow-up clinical care [37,38]. In Malawi, structural integration took the form of embedding a psychosocial assessment tool (HEADSS) into adolescent “Teen Club” HIV programs [28]. Although this showed some positive improvement in reported outcomes such as improved counseling, provider–client relationships, and potential early detection of psychosocial issues, the participants also reported high workload, space constraints and lack of appropriate training on the tool. This again underscores the importance of ensuring adequate capacity building of providers, staffing and space for better outcomes.

The scoping review also examined the impact of integration models on patient outcomes, and across the ten studies, the different models showed notable patient-level benefits. In the structural integration models [23,28] where screening was embedded in routine HIV services, there was increased early identification of common mental health disorders. In the HEADSS study in Malawi, providers perceived improvement in systematic counseling, and stronger interpersonal relationships between provider and client [28]. Early detection of mental and psychosocial issues was noted in the two studies, with 8.9% of patients screening positive for at least one mental disorder and viremia strongly associated with depression and or PTSD in [23]. This relatively low detection rate may suggest potential underreporting or missed cases within routine screening contexts.

Task-shifting emerges as a practical strategy to expand access to mental health care within resource-limited HIV systems. The task-shifting and capacity-building models [29,30,31] showed improvements in provider competencies, which resulted in increased screening from 8% to 21% post training, and resultant identification of common mental disorders. Although those patients screening positive received counseling and referral for further management, longer-term patient outcomes were not consistently reported. These findings support capacity-building models as key integration pathways, emphasizing training, mentorship, and supportive supervision as critical to effective and sustainable mental health services in low-resource HIV settings.

Additionally, group interpersonal psychotherapy delivered by trained community health workers to AYP with HIV in Kenya [29] demonstrated high acceptability and strong engagement, with most participants attending all sessions and reporting improvements in interpersonal relationships, reduced psychological distress, and reduced HIV-related stigma. This highlights the feasibility of delivering evidence-based psychological interventions through non-specialist providers in routine clinical settings.

The multi-country study examining peer-led and community-based approaches [32] and the cultural adaptation intervention study [33] both strongly highlight positive attitude from the patients due to high cultural relevance and acceptability of the approaches. Peer-led and community-anchored integration. In contrast to facility-based integration, Laurenzi et al. [32] introduces a peer-support model (ABCD) framework across four countries (Malawi, Tanzania, Uganda, and Zambia). This approach frames integration through social and community embeddedness, and positions trained young peer supporters as channels for psychosocial support and linkage to clinical care.

By embedding mental health support within adolescent peer networks, these models are likely to reduce stigma, enhance adherence, and build a sense of common resilience among young beneficiaries of HIV services. The participatory co-development process also ensures that interventions are relevant and acceptable to the context. Both studies report reductions in stigma, improved support networks, enhanced adherence, and better emotional regulation. The mindfulness and acceptance-based intervention (Discoverer, Noticer, Advisor–Values model, DNA-v) in Uganda [34] for adolescents on antiretroviral therapy (ART) helped young people strengthen coping skills, reduce unhelpful thoughts, and make positive, mindful choices. Evidence from randomized trials further suggests that mindfulness and acceptance-based interventions can improve psychological flexibility among adolescents living with HIV. While such approaches may not immediately translate into improved ART adherence, they can strengthen resilience, emotional regulation and self-acceptance, mechanisms that may support sustained engagement in care over time.

Together, these approaches illustrate evolving strategies through which health systems in resource-limited settings are addressing the dual burden of HIV and mental health among adolescents and young people.

These findings collectively demonstrate that adapting evidence-based interventions to local cultural contexts enhances uptake and sustainability. Interventions incorporating peer engagement, cultural adaptation, and community involvement appear particularly well suited to adolescent HIV care settings in LMICs.

Several potential sources of bias were observed across the included studies. Many relied on small pilot samples, qualitative designs, or single-site implementations, limiting generalizability and introducing potential selection bias. In qualitative studies, participants were often purposively selected based on their ability to articulate experiences or their involvement in adolescent HIV services, which may not fully reflect the perspectives of the broader population of adolescents and young people receiving HIV services. A further concern relates to potential under-reporting of mental health disorders in structural integration models, where mental health screening was embedded within routine HIV clinical services. For example, although one large cohort study reported high screening coverage among adolescents receiving ART, the proportion of participants screening positive for mental health conditions appeared lower than prevalence estimates reported in other studies of adolescents living with HIV. This discrepancy may reflect contextual factors such as stigma, limited mental health literacy, time constraints during routine consultations, or the use of screening tools not fully validated for adolescent populations in LMICs.

Additionally, the predominance of cross-sectional and short-term studies limits understanding of the sustainability and long-term impact of mental health–HIV integration.

As a result, while the findings provide valuable insights into emerging integration models, they also highlight important gaps in the evidence base regarding the effectiveness, scalability, and sustainability of integrated mental health–HIV services for adolescents in LMIC contexts. There is a need for longitudinal research to assess the sustainability of outcomes, retention in care, and long-term mental health and HIV-related outcomes among adolescents and young people.

**Figure 2:**
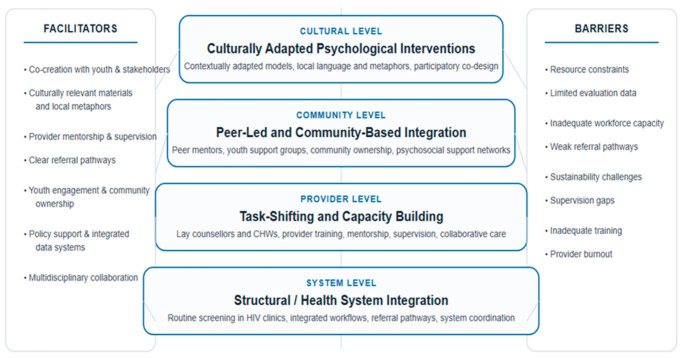
Multi-level illustration of facilitators and barriers to integrating mental health services into HIV care for adolescents and young people in LMICs.

Lastly, the scoping review sought to explore some cross cutting barriers and facilitators to integrating mental health into HIV service for AYP in LMICs. Figure 2 below summarizes these findings as they relate to the different integration models.

## 5. Conclusions

This scoping review highlights growing momentum toward integrating mental health into HIV programs for adolescents and young people in LMICs. The four dominant models: system-level integration, task-shifting, peer-led community approaches, and culturally adapted psychosocial interventions, demonstrate both innovation and feasibility across diverse contexts. Collectively, they underscore that effective HIV care must extend beyond biomedical treatment to address the mental, emotional, and social realities of young people.

It is evident that there is no single solution to addressing the complex relationship between HIV and mental health among adolescents. Facility-based screening provides critical entry points; task-shifting increases coverage and ensures service continuity; and community-anchored peer models extend psychosocial support beyond the clinic. It is clear that effective integration of mental health and HIV services for adolescents in resource-limited settings requires multi-layered and contextually adapted models. Ultimately, meaningful integration will depend on multisectoral collaboration, policy commitment, and the active participation of young people in co-creating services that reflect their lived experiences and promote holistic well-being. Future programs should pursue a mixture of strategies combining these approaches to achieve holistic, adolescent-centered care that improves both mental health and HIV outcomes.

### Limitations

Several considerations should be kept in mind when interpreting the findings of this review. Many of the included studies were qualitative or pilot in design and involved relatively small samples, which reflects the early stage of research in this area. While such approaches are valuable for exploring implementation experiences and contextual factors, future studies with larger and more diverse samples could further strengthen the evidence base. In addition, most studies focused on feasibility, acceptability, and early implementation outcomes, with limited longitudinal research examining the longer-term effects of integration models on mental health outcomes, treatment adherence, or HIV-related clinical indicators.

There was also variation across study settings, populations, and intervention approaches, including differences in countries, health system contexts, and types of mental health interventions integrated within HIV services. This diversity highlights the range of approaches currently being explored in LMIC settings; however, it also made direct comparison across studies challenging. Finally, the eligibility criteria applied in this review, particularly the focus on adolescents and young people within HIV service settings in LMICs—were intentionally specific and may have limited the number of eligible studies identified. However, this focus ensured that the evidence synthesized remained closely aligned with the aims and scope of the review.

## Figures and Tables

**Table 1:** Characteristics of included studies and integration models (*n* = 10).

Study	Countries	Study Design	Target Population	Integration Model	Reported Outcomes
Kip et al. [28]	Malawi	Qualitative (CFIR)	Providers serving adolescents with HIV (10–19)	Psychosocial screening (HEADSS) integrated into HIV care	Improved counseling and early detection of psychosocial issues
Concepcion et al. [29]	Kenya	Quasi-experimental pilot	Adolescent girls and young women (16–25)	Provider training with simulated patients for mental health screening	Screening increased (8% to 21%); improved provider communication
Yator et al. [30]	Kenya	Mixed-method pilot	Postpartum adolescents with HIV (15–24)	Group Interpersonal Therapy delivered by CHWs	Reduced depressive symptoms; improved interpersonal relationships
Petersen Williams et al. [31]	South Africa	Qualitative	Young women on ART (18–35) *	Lay health worker MI-PST counseling integrated into HIV care	Improved coping strategies; reduced alcohol use; better ART adherence
Laurenzi et al. [32]	Malawi, Tanzania, Uganda, Zambia	Participatory co-development	Young mothers living with HIV (18–24)	Peer-led psychosocial support (ABCD model)	Reduced stigma; improved peer-support networks
Harrison et al. [33], 2023	South Africa	Mixed-method pilot	Adolescents and young adults with chronic illnesses including HIV (13–24)	Clinic-based peer-support groups	Improved resilience; illness acceptance; reduced stigma
Khamisi et al. [34]	Uganda	Mixed-method adaptation	Adolescents living with HIV (10–19)	Culturally adapted ACT/mindfulness intervention	Improved emotional regulation and intervention acceptability
Musanje et al. [35]	Uganda	Randomized controlled trial	Adolescents on ART (15–19)	Group-based mindfulness and acceptance training	Improved psychological flexibility; no change in ART adherence
Njau et al. [36]	Tanzania	Qualitative (CFIR)	Adolescents and young people with HIV (11–24)	Psychological intervention integrated within HIV clinics	High acceptability; readiness for implementation
Haas et al. [23]	South Africa	Prospective cohort	Adolescents receiving ART (10–19)	Routine mental health screening integrated into ART services	Mental health conditions associated with unsuppressed viral load

* Study includes young adults beyond 24 years but was retained due to relevance to young women living with HIV. While the inclusion criteria focused on adolescents and young people aged 10–24 years, one study included young women of up to 35 years of age. This study was retained due to its strong relevance to integrated mental health and HIV service delivery among young women receiving ART.

**Table 2:** Summary of mental health–HIV integration models identified across included studies.

Integration Model	Studies	Countries	Integration Strategy	Implementation Components	Reported Outcomes
Structural/System-Level Integration	Haas et al. [23]; Kip et al. [28]	South Africa; Malawi	Integration of standardized mental health screening within routine HIV care	Use of validated screening tools (PHQ-9, GAD-7, and HEADSS) during routine HIV visits; structured referral pathways to counseling or mental health services;	Increased detection of common mental disorders; improved provider–patient communication; more structured psychosocial assessment within HIV services
Task-Shifting and Capacity Building	Concepcion et al. [29]; Yator et al. [30]; Petersen et al. [31]	Kenya; South Africa	Delivery of psychosocial interventions by trained non-specialist providers	training community health workers or lay counselors; structured supervision and mentorship; delivery of interventions such as group interpersonal therapy (IPT-G) or motivational interviewing and problem-solving therapy;	Improved provider capacity; increased screening and referral; reductions in stigma and psychological distress; improved coping skills and ART adherence behaviors
Peer-Led and Community-Based Integration	Laurenzi et al. [32]; Harrison et al. [33]	Malawi; Tanzania; Uganda; Zambia; South Africa	Peer-led psychosocial support integrated within HIV care programs	peer mentors or youth-led support groups embedded in clinic or community programs; structured group sessions addressing stigma, illness acceptance, and coping;	Reduced internalized stigma; increased resilience and social connectedness; improved attitudes toward illness; reduced depressive symptoms
Culturally Adapted Psychological Interventions	Khamisi et al. [34]; Musanje et al. [35]; Njau et al. [36]	Uganda; Tanzania	Adaptation of evidence-based psychological therapies for adolescent HIV contexts	group-based mindfulness; acceptance and commitment therapy (ACT); or DNA-v interventions tailored for adolescents on ART	Improved psychological flexibility and emotional regulation; enhanced coping skills; high intervention acceptability and feasibility in HIV care settings

## Data Availability

The data presented in this study are available upon request from the corresponding author, P.L.R.

## Supplementary Materials

The following supporting information can be downloaded at: https://www.mdpi.com/article/10.3390/ijerph23050589/s1, Table S1: Preferred Reporting Items for Systematic reviews and Meta-Analyses extension for Scoping Reviews (PRISMA-ScR) Checklist.

## Abbreviations

The following abbreviations are used in this manuscript:

ACT	Acceptance and Commitment Therapy
ART	Antiretroviral Therapy
AYP	Adolescents and Young People
DNA-V	Discoverer, Noticer, Advisor–Values
HIV	Human Immunodeficiency Virus
LMICs	Low- and Middle-Income Countries
PHQ-9	Patient Health Questionnaire-9
GAD-7	Generalized Anxiety Disorder-7
PC-PTSD-5	Primary Care Post-Traumatic Stress Disorder Screen-5
PLHIV	People Living with HIV
PRISMA-ScR	Preferred Reporting Items for Systematic Reviews and Meta-Analyses Extension for Scoping Reviews
PCC	Population–Concept–Context
SDG	Sustainable Development Goal
UNAIDS	Joint United Nations Program on HIV/AIDS
WHO	World Health Organization

## References

[1] WHO WHO Guidelines on Mental Health and Well-Being. https://www.who.int/data/gho/data/major-themes/health-and-well-being.

[2] Moitra M., Owens S., Hailemariam M., Wilson K.S., Mensa-Kwao A., Gonese G., Kamamia C.K., White B., Young D.M., Collins P.Y. (2023). Global mental health: Where we are and where we are going. Curr. Psychiatry Rep..

[3] Alloh F.T., Regmi P., Onche I., Van Teijlingen E., Trenoweth S. (2018). Mental health in low-and middle-income countries (LMICs): Going beyond the need for funding. Health Prospect. J. Pub. Health.

[4] UN Transforming Our World: The 2030 Agenda for Sustainable Development. https://sdgs.un.org/2030agenda.

[5] WHO (2022). Barriers to Mental Health Care in Africa. https://www.afro.who.int/news/barriers-mental-health-care-africa.

[6] Mayston R., Kinyanda E., Chishinga N., Prince P. (2012). Mental disorder and the outcome of HIV/AIDS in low-income and middle-income countries: A systematic review. AIDS.

[7] Collins P.Y., Velloza J., Concepcion T., Oseso L., Chwastiak L., Kemp C.G., Simoni J., Wagenaar B.H. (2021). Intervening for HIV prevention and mental health: A review of global literature. J. Int. AIDS Soc..

[8] Sherr L., Clucas C., Harding R., Sibley E., Catalan J. (2011). HIV and Depression—A systematic review of interventions. Psychol. Health Med..

[9] Ma H., Zhu F., Zhai H., Ma Y., Liu Y., Wang S., Xu Y. (2023). Prevalence of psychological distress among people living with HIV/AIDS: A systematic review and meta-analysis. AIDS Care.

[10] UNGA (1981). Report of the Advisory Committee for the International Youth Year (A/36/215).

[11] UNGA (1985). World Programme of Action for Youth (A/RES/37/52).

[12] WHO Health for the World’s Adolescents: A Second Chance in the Second Decade. https://www.who.int/publications/i/item/WHO-FWC-MCA-14.05.

[13] WHO Integrating Psychosocial Interventions and Support into HIV Services for Adolescents and Young Adults: Technical Brief. https://iris.who.int/handle/10665/369133.

[14] WHO Mental Health of Adolescents. https://www.who.int/news-room/fact-sheets/detail/adolescent-mental-health.

[15] Mei C., Fitzsimons J., Allen N., Alvarez-Jimenez M., Amminger G.P., Browne V., Cannon M., Davis M., Dooley B., Hickie I.B. (2020). Global research priorities for youth mental health. Early Interv. Psychiatry.

[16] Chantaratin S., Trimetha K., Werarak P., Lapphra K., Maleesatharn A., Rungmaitree S., Wittawatmongkol O., Phongsamart W., Kongstan N., Khumcha B. (2022). Depression and Anxiety in Youth and Young Adults Living with HIV: Frequency and Associated Factors in Thai Setting. J. Int. Assoc. Provid. AIDS Care..

[17] Buckingham E., Schrage E., Cournos F. (2013). Why the Treatment of Mental Disorders Is an Important Component of HIV Prevention among People Who Inject Drugs. Adv. Prev. Med..

[18] Grimes K.E.L., Ebasone P.V., Dzudie A., Nash D., Wainberg M.L., Pence B.W., Barrington C., Pefura E., Yotebieng M., Anastos K. (2024). Factors influencing integration of mental health screening and treatment at HIV clinic settings in Cameroon: A qualitative study of health providers’ perspectives. BMC Health Serv. Res..

[19] Wissow L.S., Tegegn T., Asheber K., McNabb M., Weldegebreal T., Jerene D., Ruff A. (2015). Collaboratively reframing mental health for integration of HIV care in Ethiopia. Health Policy Plan..

[20] Ahmed I., Weldegebreal T., Mekonnen A. (2020). Integrating mental health services into human immunodeficiency virus clinics: Lessons from task-sharing between clinical and lay healthcare providers in Ethiopia. Ethiop. J. Health Dev..

[21] Conteh N.K., Latona A., Mahomed O. (2023). Mapping the effectiveness of integrating mental health in HIV programs: A scoping review. BMC Health Serv. Res..

[22] Kabunga A., Namata H., Kigongo E., Musinguzi M., Tumwesigye R., Auma A.G., Nabaziwa J., Shikanga E.M., Okalo P., Nalwoga V. (2024). Exploring effective approaches: Integrating mental health services into HIV clinics in Northern Uganda. HIV/AIDS-Res. Palliat. Care.

[23] Haas A.D., Technau K.G., Pahad S., Braithwaite K., Madzivhandila M., Sorour G., Sawry S., Maxwell N., von Groote P., Tlali M. (2020). IeDEA Southern Africa Collaboration. Mental health, substance use and viral suppression in adolescents receiving antiretroviral therapy at a paediatric HIV clinic in South Africa. AIDS.

[24] Fabian K.E., Muanido A., Cumbe V.F.J., Mukunta C., Manaca N., Dorsey S., Hammett W.H., Wagenaar B.H. (2021). Integrating a transdiagnostic psychological intervention into routine HIV care: A mixed-methods evaluation of the Common Elements Treatment Approach in Mozambique (CETA-MZ). BMC Health Serv. Res..

[25] Poku O.B., Stewart N.S., Kuo C., LeGrand E.L., Dowdy D.W., Murray S.M., Bass J.K. (2023). Mental health problems among adolescents living with HIV across the HIV care continuum in sub-Saharan Africa: A scoping review. AIDS Behav..

[26] Tricco A.C., Lillie E., Zarin W., O’Brien K.K., Colquhoun H., Levac D., Moher D., Peters M.D.J., Horsley T., Weeks L. (2018). PRISMA extension for scoping reviews (PRISMA-ScR): Checklist and explanation. Annals Inter. Med..

[27] Munn Z., Peters M.D.J., Stern C., Tufanaru C., McArthur A., Aromataris E. (2018). Systematic review or scoping review? Guidance for authors when choosing between a systematic or scoping review approach. BMC Med. Res. Methodol..

[28] Kip E.C., Udedi M., Kulisewa K., Go V.F., Gaynes B.N. (2022). Barriers and facilitators to implementing the HEADSS psychosocial screening tool for adolescents living with HIV/AIDS in teen club programs in Malawi: Health care providers’ perspectives. BMC Health Serv. Res..

[29] Concepcion T., Mogere P., Ngure K., Mwathi N., Njiru R., Kipkorir B., Kiptinness C., Maina G., Owidi E., Owens T. (2023). Higher rates of mental health screening of adolescents recorded after provider training using simulated patients in a Kenyan HIV clinic: Results of a pilot study. Front. Pub. Health.

[30] Yator O., Khasakhala L., Stewart G.J. (2022). Acceptability and impact of group interpersonal therapy (IPT-G) on Kenyan adolescent mothers living with human immunodeficiency virus (HIV): A qualitative analysis. BMC Women’s Health.

[31] Petersen-Williams P., Brooke-Sumner C., Joska J., Kruger J., Vanleeuw L., Dada S., Sorsdahl K., Myers B. (2020). Young South African women on antiretroviral therapy perceptions of a psychological counselling program to reduce heavy drinking and depression. Int. J. Environ. Res. Public Health.

[32] Laurenzi C., Ronan A., Phillips L., Nalugo S., Mupakile E., Operario D., Toska E. (2023). Enhancing a peer supporter intervention for young mothers living with HIV in Malawi, Tanzania, Uganda, and Zambia: Adaptation and co-development of a psychosocial component. Glob. Public Health.

[33] Harrison A., Mtukushe B., Kuo C., Wilson-Barthes M., Davidson B., Sher R., Galárraga O., Hoare J. (2023). Better Together: Acceptability, feasibility and preliminary impact of chronic illness peer support groups for South African adolescents and young adults. J. Int. AIDS Soc..

[34] Khamisi M., Camlin C.S., Kamya M.R., Vanderplasschen W., Sinclair D.L., Getahun M., Kirabo H., Nangendo J., Kiweewa J., White R.G. (2023). Culturally adapting a mindfulness and acceptance-based intervention to support the mental health of adolescents on antiretroviral therapy in Uganda. PLoS Glob. Public Health.

[35] Musanje K., Kamya M.R., Kasujja R., Vanderplasschen W., Sinclair D.L., Baluku M.M., Odokonyero R.F., Namisi C.P., Mukisa J., White R.G. (2024). The effect of a group-based mindfulness and acceptance training on psychological flexibility and adherence to antiretroviral therapy among adolescents in Uganda: An open-label randomized trial. J. Int. Assoc. Provid. AIDS Care.

[36] Njau T., Mwakawanga D.L., Sunguya B., Minja A., Kaaya S., Fekadu A. (2024). Perceived barriers and opportunities for implementing an integrated psychological intervention for depression in adolescents living with HIV in Tanzania. BMC Health Serv. Res..

[37] Rutakumwa R., Ssebunnya J., Mugisha J., Mpango S., Tusiime R., Kyohangirwe C., Taasi L., Sentongo G., Kaleebu H., Patel V. (2021). Stakeholders’ perspectives on integrating the management of depression into routine HIV care in Uganda: Qualitative findings from a feasibility study. Int. J. Ment. Health Syst..

[38] UNAIDS Integrating Mental Health and HIV Services: Key Considerations. Joint United Nations Programme on HIV/AIDS. https://www.unaids.org/en/resources/presscentre/featurestories/2022/april/20220428_integrate-hiv-mental-health.

