# Supplementary material for: Models of Integration for Mental Health and HIV/AIDS Among Adolescents and Young People in Low- and Middle-Income Countries: A Scoping Review
Source: Int J Environ Res Public Health. 2026 Apr 30;23(5):589. doi: 10.3390/ijerph23050589 (PMC13206699; doi:10.3390/ijerph23050589)
Supplement: Supplementary file 1 [file ijerph-23-00589-s001.zip › ijerph-4173135-supplementary.pdf]

Table S1. Preferred Reporting Items for Systematic reviews and Meta-Analyses extension for Scoping Reviews (PRISMA-ScR) Checklist

SECTION	ITEM	PRISMA-ScR CHECKLIST ITEM	REPORTED ON PAGE #
**TITLE**			
**Title**	1	**Models of Integration for Mental Health and HIV/AIDS among adolescents and Young People in Low- and Middle-Income Countries: A Scoping Review**	1
**ABSTRACT**			
**Structured summary**	2	Adolescents and young people (AYP) experience a disproportionate burden of both mental health conditions and HIV, particularly in low- and middle-income countries (LMICs). Mental health problems such as depression, anxiety, and substance use increase HIV risk and negatively affect treatment adherence and outcomes, yet mental health remains insufficiently integrated into HIV research and programming. Evidence on how mental health services are operationally integrated into HIV prevention and treatment for this population is limited and fragmented. This scoping review mapped existing evidence on the integration of mental health services into HIV treatment programs for AYP in LMICs. Guided by PRISMA-ScR and the Person–Concept–Context framework, PubMed and PsycINFO were searched for studies published between 2014 and 2024. Eligible studies reported mental health screening, assessment, treatment, or referral within HIV services for AYP in LMICs. Two reviewers independently screened studies, assessed full texts, and extracted data. Of 556 records identified, six studies met the inclusion criteria. All were conducted in sub-Saharan Africa and primarily used qualitative or pilot designs. Four integration approaches were identified: routine mental health screening within HIV services, task-shifting to trained lay providers, peer-led and community-based psychosocial support, and culturally adapted, youth-centered psychological interventions. Common barriers included stigma, low mental health literacy, limited training and supervision, staffing constraints, and weak referral systems. Though existing evidence remains preliminary, integration of mental health into HIV care for adolescents in LMICs appears feasible and acceptable when contextually adapted and participatory.	2
**INTRODUCTION**			

**Rationale**	3	Mental health is a fundamental component of the World Health Organization's definition of health as a state in which an individual is free from any physical, mental and social illnesses or disorders; and is able to adequately handle daily life challenges as an active member of society[**Error! Reference source not found.**].<br>Globally, more than one in ten adolescents globally are affected by a mental disorder, and about 40% of these are anxiety or depressive disorders [**Error! Reference source not found.**]. Despite being globally recognized as an important public health issue, mental health is still less prioritized as a disease burden in many Low-and Middle-Income Countries (LMICs and yet more than 70% of the global mental health burden occurs in these countries [**Error! Reference source not found.**].<br>According to the United Nations Development Agenda, Sustainable Development Goal (SDG) 3 aims to ensure healthy lives and promote well-being for all at all ages [**Error! Reference source not found.**]. This goal acknowledges that good health is crucial for individuals to lead full, productive lives and for societies to flourish. The agenda includes targets to end communicable diseases like HIV/AIDS, preventing and treating non-communicable diseases and mental health and strengthening health systems to achieve universal health coverage. Therefore, addressing HIV, mental health and related substance abuse is not only a more effective and sustainable approach and health priority, but also a powerful driver for achieving SDG 3— creating healthier individuals, stronger communities, and more resilient health systems. Most African countries bear the bigger burden of HIV/AIDS and have competing health and development priorities with insufficient funds to adequately address mental health conditions. As a result, mental healthcare is severely underfunded. Data recorded in health management systems do not include mental health, which contributes to an underappreciation of the disease burden in countries across the continent [**Error! Reference source not found.**]. Lack of data means that policymakers cannot comprehend the depth of the problem that countries are facing.<br>The relationship between mental health and HIV is complex and bidirectional. Poor mental health serves as a risk factor for HIV infection by influencing high-risk behaviors, worsening disease progression, and reducing adherence to treatment. Conversely, living with HIV often leads to or intensifies mental health conditions such as depression, anxiety, trauma, and substance use disorders [**Error! Reference source not found.**, **Error! Reference source not found.**, **Error! Reference source not found.**, in **Error! Reference source not found.**].<br>Adolescence represents a crucial period of growth and development, marked by significant psychological, social, and biological transitions [**Error! Reference source not found.**] The age category 'youth 'or 'young person' tends to be fluid across countries and socio-cultural and economic contexts. Many countries use the term 'young person' and 'adolescent' interchangeably depending on the context. The United Nations defines 'youth' as anyone between the ages of 15-24 years [**Error! Reference source not found.**,**Error! Reference source not found.**]. The World Health Organization also defines 'Adolescents' as persons aged 10-19 years, 'Youth' as the age -range 15–24, and 'Young People' as the age range 10-24 years [**Error! Reference source not found.**] .This means there are definition overlaps on the age range 15-19 old people who can be classified as adolescents and young people; however these definitions do not override what countries may ultimately choose to adopt as working definitions for their contexts. For the purpose of this review, adolescents and young people (AYP) refer to individuals aged 10–24 years—a population whose health outcomes are vital to long-term community well-being.<br>During adolescence, individuals are especially vulnerable to mental health problems and to behaviors that increase the risk of HIV infection. Globally, the prevalence of mental health disorders among adolescents continues to rise. There is evidence that 34.6% of mental health disorders begin as early as 14 years of age, and up to 62.5% by the age of 25[**Error! Reference source not found.**].	3

		According to the WHO's Mental Health of Adolescents Factsheet, one in seven adolescents aged 10–19 years experiences a mental disorder, accounting for about 13% of the global disease burden in this age group[**Error! Reference source not found.**]. Suicide remains the fourth leading cause of death among young people aged 15–29 years[**Error! Reference source not found.**,**Error! Reference source not found.**]. Adolescents living with HIV generally have a higher prevalence of mental health conditions (e.g. depression and anxiety) compared with their HIV-negative peers[**Error! Reference source not found.**]. Treating comorbid mental illnesses such as depression, anxiety, and substance use disorders can improve adherence to care and clinical outcomes for PLHIV. These figures underscore the urgent need for focused interventions addressing both mental health and HIV among young people.<br>Untreated mental health problems—particularly depression and substance use—are linked to increased HIV risk behaviors, reduced access to care, poor adherence to antiretroviral therapy (ART), and higher morbidity and mortality related to HIV[**Error! Reference source not found.**]. Despite growing evidence of these interconnections, mental health needs among people living with or at risk of HIV remain largely unmet, particularly in low- and middle-income countries (LMICs). In most health systems, mental health services are typically accessed only when symptoms become severe, or a formal diagnosis has been made [**Error! Reference source not found.**]<br><br>Existing studies and interventions integrating mental health and HIV services vary across settings, target populations, and service delivery models. Much of the existing literature and previous reviews have primarily focused on general populations[**Error! Reference source not found.**,**Error! Reference source not found.**,**Error! Reference source not found.Error! Reference source not found.**,**Error! Reference source not found.**] and/or the burden of mental health conditions among those living with HIV[**Error! Reference source not found.**,**Error! Reference source not found.**,**Error! Reference source not found.**] rather than examining how mental health services are operationally integrated into HIV care. Consequently, there is limited synthesis of evidence specifically addressing integrated service delivery models for adolescents and young people, particularly in low- and middle-income countries (LMICs). This scoping review seeks to address these gaps by systematically mapping and synthesizing available evidence on models of mental health and HIV service integration for adolescents and young people in LMICs.<br>A scoping review was identified as the most suitable method for exploring this topic, as it allowed for mapping the breadth and depth of available evidence, identifying intervention models, and highlighting research gaps[**Error! Reference source not found.**]. The review focused on integration models used in LMICs and examined their benefits on the wellbeing of adolescents and young people. The purpose of this scoping review was to map and synthesize existing evidence on the integration of mental health services into HIV prevention and treatment programs for adolescents and young people in LMICs.	
**Objectives**	4	**Provide an <u>explicit statement of the questions and objectives being</u> addressed with reference to their key elements (e.g., population or participants, concepts, and context) or other relevant key elements used to conceptualize the review questions and/or objectives.**<br><br>The review was conducted in line with the Joanna Briggs Institute (JBI) methodology for scoping reviews and is reported according to the PRISMA Extension for Scoping Reviews (PRISMA-ScR)[**Error! Reference source not found.**]. The review followed the recommended JBI steps, including formulation of the review questions using the Population–Concept–Context framework, development and implementation of a systematic search strategy, independent screening and selection of studies, data extraction using a predefined charting tool, and synthesis and presentation of findings in a transparent and reproducible manner.	4

		The eligibility criteria and review questions were developed using the Person–Concept–Context (PCC) where the population (Person) comprised adolescents and young people receiving HIV prevention or treatment services; the concept focused on the integration of mental health interventions, including screening, assessment, treatment, or referral, within HIV care; and the context was limited to low- and middle-income countries (LMICs). The purpose of this scoping review was to map and synthesize existing evidence on the integration of mental health services into HIV prevention and treatment programs for adolescents and young people in LMICs. This is the first review to review to:<br>1. Identify models of integration between mental health and HIV services implemented for adolescents and young people in LMICs.<br>2. Examine how these integration models influenced outcomes in this target population<br>3. Identify the facilitators and barriers reported in implementing different integration approached for mental health and HIV	
**METHODS**			
**Protocol and registration**	5	**Indicate whether a review protocol exists; state if and where it can be accessed (e.g., a Web address); and if available, provide registration information, including the registration number.**<br><br>The protocol for this scoping review is part of the overall study that was approved by the Health Sciences Research Ethics Committee (HSREC) of the University of Free State (Ref: UFS-HSD2024/1691/2502) The scoping review was registered with Open Science Framework (OSF), with the registration DOI: https://doi.org/10.17605/OSF.IO/VB2J7.	5
Eligibility criteria	6	**Specify characteristics of the sources of evidence used as eligibility criteria (e.g., years considered, language, and publication status), and provide a rationale.**<br><br>This scoping review will include studies that report on mental health interventions—such as screening, assessment, treatment, or referral—delivered to adolescents and young people receiving HIV prevention or treatment services in low- and middle-income countries (LMICs). Only studies published in English and from 2014 onward will be considered, ensuring the inclusion of contemporary evidence.<br>**Inclusion Criteria**<br>Studies were included if they met the following conditions:<br>• Reported on a mental health intervention—including screening, assessment, treatment, or referral—implemented among adolescents and young people aged 10–24 years.<br>• The intervention was delivered within HIV prevention or treatment services.<br>• The study was conducted in a low- or middle-income country (LMIC) setting.<br>• Published in the English language.<br>• Published from 2014 onwards, to capture recent developments and contemporary evidence in service integration.<br><br>**Exclusion Criteria**<br>Studies were excluded if they met any of the following conditions:<br>• Conducted before 2014 or outside LMIC settings.<br>• Focused on HIV prevention or treatment services for the general population rather than specifically on adolescents and young people.	5

		• Implemented in non-healthcare settings, such as schools or community programs not linked to formal health services.<br>• Did not include any component of mental health intervention within HIV-related care.	
Information sources*	7	**Describe all information sources in the search (e.g., databases with dates of coverage and contact with authors to identify additional sources), as well as the date the most recent search was executed.**<br><br>In addition to a librarian-assisted search, a comprehensive literature search was conducted across selected scientific databases, including PsycINFO and PubMed, to identify relevant studies. The search strategy combined both index terms and free-text keywords to capture the breadth of evidence on the integration of mental health into HIV prevention and treatment services for adolescents and young people in low- and middle-income countries (LMICs). The search terms were formulated based on the review questions. The search was limited to studies published in the English language and from 2014 onwards, in accordance with the inclusion criteria. Reference lists and citations of all primary articles retrieved during the initial search were also screened to identify additional relevant studies not captured in the database searches and searched through Goggle Scholar. The following key search terms and index words were used individually and in different combinations with Boolean operators (AND/OR): “HIV”, AND “mental health”; “Youth” OR “Adolescent mental health”; “HIV AND mental health integration”; “Integration models” and “Adolescent HIV AND mental health”	5
Search	8	**Present the full electronic search strategy for at least 1 database, including any limits used, such that it could be repeated.**<br><br>A comprehensive electronic search was conducted in PubMed to identify relevant studies examining the integration of mental health services into HIV prevention and treatment programs for adolescents and young people in low- and middle-income countries (LMICs). The search strategy combined Medical Subject Headings (MeSH) and free text terms and was developed in consultation with a librarian. Boolean operators (AND/OR) were used to combine concepts related to HIV, mental health, adolescents and young people, and service integration.	5
Selection of sources of evidence	9	**State the process for selecting sources of evidence (i.e., screening and eligibility) included in the scoping review.**<br><br>The Rayyan The review and selection of the articles was done by two independent reviewers using an agreed upon inclusion criteria to reduce errors and potential bias. The reviewers then came together to compare the independent lists and further assess full articles and abstracts for eligibility. Articles that met the agreed upon inclusion criteria were selected and kept for inclusion, while removing any duplicate records in the system.	6

<table>
<tr>
<td>Data charting process</td>
<td>10</td>
<td>Describe the methods of charting data from the included sources of evidence (e.g., calibrated forms or forms that have been tested by the team before their use, and whether data charting was done independently or in duplicate) and any processes for obtaining and confirming data from investigators.

The PI developed a data charting template to capture key characteristics of the selected studies and the critical information that is relevant to the objectives of the scoping review. The charting tool was designed to capture the following data:

• Title
• Publication Year
• Objective/s
• Methodology
• Target population
• Integration model
• Location/Setting
• Key findings as they relate to the scoping review questions</td>
<td>7</td>
</tr>
<tr>
<td>Data items</td>
<td>11</td>
<td>List and define all variables for which data were sought and any assumptions and simplifications made.
The following assumptions were made in extracting information for the sources:
• Studies describing populations aged 10–24 years, were categorized as youth even if the term ‘adolescent’ and or young people was not used.
• Combining terms like “mental health services”, “psychosocial support”, and “counselling” under one variable if they served a similar function.
• Simplifying integration models into broad categories (e.g., “fully integrated,” “partially integrated,” “linked/referral-based”).
Data charting tool:
<table>
<tr><th></th><th>Article 1</th><th>Article 2</th><th>Article 3</th><th>Article 4</th></tr>
<tr><td>Citation (Title, Author, Year)</td><td></td><td></td><td></td><td></td></tr>
<tr><td>Objective</td><td></td><td></td><td></td><td></td></tr>
<tr><td>Methodology/Design</td><td></td><td></td><td></td><td></td></tr>
<tr><td>Target population</td><td></td><td></td><td></td><td></td></tr>
<tr><td>Location/Setting</td><td></td><td></td><td></td><td></td></tr>
<tr><td>Intervention/Integration model/s</td><td></td><td></td><td></td><td></td></tr>
<tr><td>Key findings, including reported outcomes, barriers, and facilitators</td><td></td><td></td><td></td><td></td></tr>
</table>
</td>
<td>7</td>
</tr>
</table>

Critical appraisal of individual sources of evidence§	12	**If done, provide a rationale for conducting a critical appraisal of included sources of evidence; describe the methods used and how this information was used in any data synthesis (if appropriate).**<br><br>Not done	
Synthesis of results	13	**Describe the methods of handling and summarizing the data that were charted.**<br>The findings of this scoping review were synthesized and tabulated narrative approach to map existing models of integration between mental health and HIV services for AYP. Extracted information included type of integration model, country, target population, service delivery setting, and reported patient-level outcomes.<br>Table was used to guide comparison across studies. Integration approaches were categorized into thematic model types based on similarities in implementation strategies, including system-level integration, task-shifting and capacity building, peer-led and community-based approaches, and culturally adapted psychosocial interventions.<br>This narrative synthesis addressed the objectives of the scoping review by:<br>(i) identifying existing models of mental health–HIV service integration implemented for adolescents and young people in LMICs;<br>(ii) examining how these integration models influenced patient- and service-level outcomes, such as feasibility, acceptability, service uptake, and early detection of mental health conditions; and<br>(iii) synthesizing reported facilitators and barriers affecting implementation across different contexts**.**	7-11

SECTION	ITEM	PRISMA-ScR CHECKLIST ITEM	REPORTED ON PAGE #
**RESULTS**			
Selection of sources of evidence	14	**Give numbers of sources of evidence screened, assessed for eligibility, and included in the review, with reasons for exclusions at each stage, ideally using a flow diagram.**<br>A total of five hundred and fifty-six (556) sources selected and uploaded into Rayyan. Of these, one hundred and forty-seven (147) duplicates were detected, while three (3) duplicates were resolved, one hundred and forty-four (144) were deleted leaving four hundred and twelve (412) articles for screening. Of the four hundred and twelve (412) selected articles, four hundred (400) did not meet the inclusion criteria and were excluded for various reasons including, but not limited to: ineligible context, study populations, or objective among others. Twelve (12) articles met the inclusion criteria and proceeded to the next level. After full text screening, six articles were dropped, and only six (6) articles were selected to go through data charting and extraction.	6

		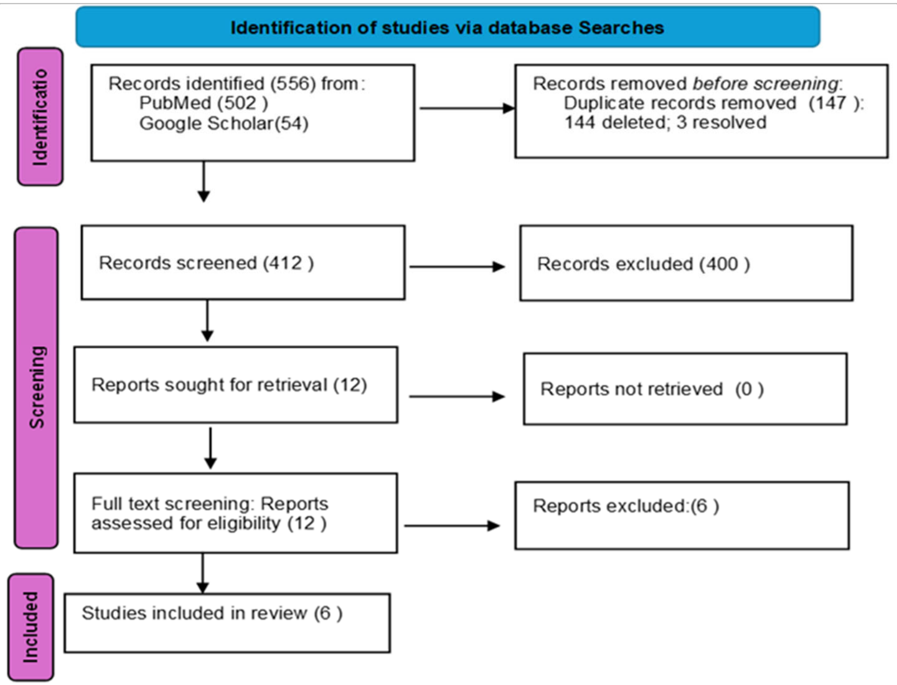	
Characteristics of sources of evidence	15	**For each source of evidence, present characteristics for which data were charted and provide the citations**.<br><br>Annex	
Critical appraisal within sources of evidence	16	Not done	
Results of individual sources of evidence	17	**For each included source of evidence, present the relevant data that were charted that relate to the review questions and objectives.**<br><br>Data were charted using a standardized extraction form. The following variables were collected from each included study: author, year, country, study design, target population, setting, intervention or integration model, mental health and HIV components, reported outcomes, facilitators, and barriers. For consistency, the term “adolescents and young people (AYP)” was applied to participants aged 10–24 years, even when studies used alternative terms such as “youth.”	

Synthesis of results	18	**Summarize and/or present the charting results as they relate to the review questions and objectives.**	
**DISCUSSION**			
Summary of evidence	19	**Summarize the main results (including an overview of concepts, themes, and types of evidence available), link to the review questions and objectives, and consider the relevance to key groups.**<br><br>The studies reviewed together highlight diverse but convergent approaches to integrating mental health services within HIV care for adolescents and young people in resource-limited settings, highlighting different, and similar themes, facilitators and barriers to integrated mental health-HIV care. Across these studies, integration was operationalized through a variety of models—ranging from structural and system-level adaptations to task-shifting, peer-led, and culturally adapted psychosocial approaches. Although *structural integration models* demonstrated success in identifying common mental health conditions, key considerations for this approach include structured and continuous capacity building and supervision for health care providers. The facility also needs to be adequately staffed as the integrated screening can increase the workload, particularly of the screening tolls are long. Addressing structural barriers such as private space for screening, patient flow, staff workload, leadership support and engagement remains key to the successful structural integration model[**Error! Reference source not found.**]. Some of these barriers, especially patient factors, are supported by Rutakumwa et. al. [**Error! Reference source not found.**] where longer patient waiting times undermined the benefits of structural integration in under-staffed settings. High screening coverage alone and increased identification of mental health conditions without further treatment is neither useful nor ethical. Unclear referral pathways in this models not only undermine the effort and impact of the screening, but also bear ethical implications of failure to provide treatment where indicted.<br><br>According to the UNAIDS guidance on mental health and HIV integration, screening must be accompanied by access to diagnostic assessment and relevant follow up clinical care [**Error! Reference source not found.**]. In Malawi, structural integration took the form of embedding a psychosocial assessment tool (HEADSS) into adolescent "Teen Club" HIV programs[**Error! Reference source not found.**]. Although this showed some positive improvement in outcomes such as improved counseling, provider–client relationships, and potential early detection of psychosocial issues, the participants expressed among other things high workload frustrations, space constraints and lack of appropriate training on the tool. This again underscores the importance of ensuring adequate capacity building of providers, staffing and space for better outcomes.<br><br>The other question the scoping review aimed to address was the *impact of integration models on patient outcomes,* and across the six studies, the different models showed notable patient level benefits. In the structural integration models [**Error! Reference source not found.**,**Error! Reference source not found.**] where screening was embedded in routine HIV services, there was reported increase in early identification of common mental health disorders. In the HEADSS study in Malawi, providers perceived improvement in systematic counseling, and stronger interpersonal relationships between provider and client [**Error! Reference source not found.**]. Early detection of mental and psychosocial issues was noted in the two studies, with 8.9% of patients screening positive for at least one mental disorder and viremia strongly associated with depressions and or PTSD in [**Error! Reference source not found.**]. Given the high prevalence of metal health disorders among AYP, especially those living with HIV, this finding may indicate under reporting.	

		Task-shifting emerges as a practical strategy to expand access to mental health care within resource-limited HIV systems. The *task-shifting and capacity building models* as demonstrated by Concepcion et al.[**Error! Reference source not found.**] (Kenya) and Njau et al. [**Error! Reference source not found.**] (Tanzania) showed improvements in provider competencies, which resulted in increased screening from 8% to 21% post training, and resultant identification of common mental disorders. Although those screening positive received counselling and referral for further management, final outcomes were not clearly documented. These findings support capacity-building models as key integration pathways, emphasizing training, mentorship, and supportive supervision as critical to effective and sustainable mental health services in low-resource HIV settings.<br><br>The multi-country study that addressed *Peer-led & community-based approaches* [**Error! Reference source not found.**] and the cultural adaptation intervention study[**Error! Reference source not found.**] both strongly highlight positive attitude from the patients, due to high cultural relevance and acceptability of the approaches. Peer-Led and Community-Anchored Integration in contrast to facility-based integration, Laurenzi et al.[**Error! Reference source not found.**] introduces a peer-support model (*ABCD)* framework—across four countries (Malawi, Tanzania, Uganda, Zambia). This approach frames integration through social and community embeddedness, and positions trained young peer supporters as channels for psychosocial support and linkage to clinical care. By embedding mental health support within adolescent peer networks, these models are likely to reduce stigma, enhance adherence, and build a sense of common resilience among young beneficiaries of HIV services. The participatory co-development process also ensures that interventions are relevant and acceptable to the context. Resultantly, these studies both report reduction in stigma, intervention acceptability, stronger support networks, improvements in adherence and emotional regulation as a result. The mindfulness and acceptance-based intervention (Discoverer, Noticer, Advisor- values model -DNA-v) in Uganda [**Error! Reference source not found.**] for adolescents on antiretroviral therapy (ART) helped young people strengthen coping skills, reduce unhelpful thoughts, and make positive, mindful choices. This demonstrates that adapting foreign concepts to local and cultural contexts, enhances uptake and ensures sustainability.<br><br>Although across these studies there are notable positive patient outcomes, there is limited documentation of long-term impact of these interventions. Again, there was no direct link of the interventions with the clinical outcomes of the patients.<br>Lastly, the scoping review sought to explore some cross cutting *barriers and facilitators* to integrating mental health into HIV service for AYP in LMICs.	
Limitations	20	**Discuss the limitations of the scoping review process**.<br>Eligibility criteria may have been too stringent in terms of setting and target population, thus limited the number of eligible studies for this review. However, this was highly relevant for the aims of the scoping review.	13
Conclusions	21	**Provide a general interpretation of the results with respect to the review questions and objectives, as well as potential implications and/or next steps.**<br>This scoping review highlights growing momentum toward integrating mental health into HIV programs for adolescents and young people in LMICs. The four dominant models: system-level integration, task-shifting, peer-led community approaches, and culturally adapted psychosocial interventions, demonstrate both innovation and feasibility across diverse contexts. Collectively, they	13

		underscore that effective HIV care must extend beyond biomedical treatment to address the mental, emotional, and social realities of young people.<br>It is evident that there is no single solution to addressing the complex relationship between HIV and mental health among adolescents. Facility-based screening provides critical entry points; task-shifting increases coverage and ensures service continuity; and community-anchored peer models extend psychosocial support beyond the clinic. It is clear that effective integration of mental health and HIV services for adolescents in resource limited settings requires *multi-layered, contextually adapted models.*<br>Ultimately, meaningful integration will depend on multisectoral collaboration, policy commitment, and the active participation of young people in co-creating services that reflect their lived experiences and promote holistic well-being. Future programs should pursue a mixture of strategies combining these approaches to achieve holistic, adolescent-centered care that improves both mental health and HIV outcomes.	
**FUNDING**			
Funding	22	**Describe sources of funding for the included sources of evidence, as well as sources of funding for the scoping review. Describe the role of the funders of the scoping review.**<br><br>No external funding was received for this review.	14

JBI = Joanna Briggs Institute; PRISMA-ScR = Preferred Reporting Items for Systematic reviews and Meta-Analyses extension for Scoping Reviews.
* Where *sources of evidence* (see second footnote) are compiled from, such as bibliographic databases, social media platforms, and Web sites.
† A more inclusive/heterogeneous term used to account for the different types of evidence or data sources (e.g.,quantitative and/or qualitative research, expert opinion, and policy documents) that may be eligible in a scoping review as opposed to only studies. This is not to be confused with *information sources* (see first footnote).
‡ The frameworks by Arksey and O'Malley (6) and Levac and colleagues (7) and the JBI guidance (4, 5) refer to theprocess of data extraction in a scoping review as data charting.
§ The process of systematically examining research evidence to assess its validity, results, and relevance before using it to inform a decision. This term is used for items 12 and 19 instead of "risk of bias" (which is more applicable to systematic reviews of interventions) to include and acknowledge the various sources of evidence that may be used in a scoping review (e.g., quantitative and/or qualitative research, expert opinion, and policy document).

From: Tricco AC, Lillie E, Zarin W, O'Brien KK, Colquhoun H, Levac D, et al. PRISMA Extension for Scoping Reviews (PRISMA-ScR): Checklist and Explanation. Ann Intern Med. ;169:467–473. doi: 10.7326/M18-0850

Annex : Characteristics of sources of evidence (15)

Citation (Title, Author, Year)	Region / Country	Objective	Methodology	Target Study Population	Integration Model/s	Patient Outcomes (Reported)
Kip et al. (2022) – Barriers and facilitators to implementing the HEADSS psychosocial screening tool for adolescents living with HIV/AIDS in Malawi	Malawi	Assess barriers and facilitators to implementing the HEADSS tool in adolescent HIV programs.	Qualitative study using semi-structured interviews guided by the CFIR framework.	20 healthcare providers involved in **adolescent ART programs** in Teen Clubs.	Implementation science (CFIR) framework for integrating psychosocial screening into HIV care.	Improved counseling, provider–client relationships, and potential early detection of psychosocial issues.
Laurenzi et al. (2023) – Enhancing a peer supporter intervention for young mothers living with HIV in Malawi, Tanzania, Uganda, and Zambia	Malawi, Tanzania, Uganda, Zambia	Adapt and co-develop a psychosocial peer-support model ('Boost') to improve young mothers' mental health.	Participatory co-development (formative research,	**Young mothers (18–24),** peer supporters, and technical advisors.	Peer-led ABCD model (Ask–Boost–Connect–Discuss) integrated with existing peer-support networks (PATA).	Feasible and acceptable; improved knowledge, reduced stigma, and stronger support networks.
Concepcion et al. (2023) – Higher rates of mental health screening of adolescents recorded after provider training using simulated patients in a Kenyan HIV clinic	Kenya	Evaluate impact of simulated patient encounter (SPE) training on CMD screening in HIV care.	Pilot quasi-experimental study with interrupted time series analysis.	**Adolescent girls and young women** (16–25) and 10 trained healthcare providers.	Simulated patient encounter model to integrate CMD screening into routine HIV care.	CMD screening increased from 8% to 21%; improved provider communication and referral practices.
Haas et al. (2020) – Mental health, substance use, and viral suppression in adolescents receiving ART at a pediatric HIV clinic in South Africa	South Africa	Examine outcomes of routine mental health screening and its association with viral suppression.	Prospective cohort study using validated screening tools (PHQ-9, GAD-7, CAGE-AID, PC-PTSD-5).	**Adolescents (10–19 years)** on ART in Johannesburg clinic.	Routine integration of mental health screening within pediatric ART programs.	8.9% screened positive; depression/PTSD/substance use linked to unsuppressed viral load; feasible screening integration.

Njau et al. (2024) – Perceived barriers and opportunities for implementing an integrated psychological intervention for depression in adolescents living with HIV in Tanzania	Tanzania (Dar es Salaam)	Explore barriers and facilitators to implementing integrated psychological care for adolescents living with HIV and depression.	Qualitative study using CFIR framework; in-depth interviews	**Adolescents (11–24),** caregivers, clinicians, and nurse counselors.	Integration of psychological interventions within HIV Care and Treatment Centers (HIV-CTCs).	High acceptability and perceived feasibility of integrated care; readiness for change among stakeholders.
Laurenzi et al. (2023) – Culturally adapting a mindfulness and acceptance-based intervention to support mental health of adolescents on ART in Uganda	Uganda	Culturally adapt an ACT and mindfulness-based intervention to support adolescents' mental health and ART adherence.	Mixed-methods adaptation process with stakeholder engagement and iterative cultural tailoring.	**Adolescents living with** HIV on ART.	Culturally adapted ACT/mindfulness sessions delivered by trained lay providers within HIV care.	Improved cultural relevance, emotional regulation, and intervention acceptability.

Data items:

When you *“list and define all variables for which data were sought,”* you’re being asked to **state exactly what information you extracted from each study** — i.e., what you looked for and recorded when assessing each included article.These variables are also sometimes called **data items**, **charting fields**, or **data extraction elements**.

🔹 Typical Variables in a Scoping Review

In your review on *integration of mental health into HIV programs for adolescents and young people*, the main variables (data items) would likely include:

Variable	Definition / Description
**Citation**	Author(s), year of publication, and source (e.g., journal name).
**Study Title / Objective**	The stated aim or purpose of the study.
**Study Design / Methodology**	Type of study (e.g., cross-sectional, RCT, qualitative, mixed methods).
**Country / Setting**	Where the study took place (e.g., LMIC context, urban vs. rural, health facility type).
**Population / Target Group**	Description of participants (e.g., adolescents aged 10–24, HIV-positive youth, key populations).
**Intervention / Integration Model**	Type of integration approach (e.g., task-sharing, co-location, psychosocial support, referral systems).

Variable	Definition / Description
**Mental Health Component**	Type of mental health service (e.g., screening, counselling, medication, therapy).
**HIV Component**	Whether the study focused on HIV prevention, treatment, or care.
**Outcomes Reported**	Measurable effects (e.g., ART adherence, mental health improvement, stigma reduction).
**Facilitators**	Factors that supported integration success (e.g., staff training, policy support).
**Barriers / Challenges**	Factors that hindered implementation (e.g., stigma, resource constraints).
**Key Findings / Conclusions**	Summary of main results and implications.
**Funding / Implementing Organization** *(optional)*	Who supported or implemented the intervention.

◆ Assumptions and Simplifications

This part means:
If you made **any assumptions** while charting data (e.g., interpreting unclear terms, grouping categories) or **simplified variables** for analysis, you should report them transparently.

Examples for your review might include:

- Assuming that studies describing *"youth"* populations referred to individuals aged **10–24 years**, even if exact ages weren't specified.
- Combining terms like *"mental health services"*, *"psychosocial support"*, and *"counselling"* under one variable if they served a similar function.
- Simplifying integration models into broad categories (e.g., *"fully integrated," "partially integrated," "linked/referral-based"*).
- Assuming *facility-based programs* include primary healthcare and hospital settings unless otherwise stated.

◆ Example Text You Could Use in Your Methods Section

> Data were charted using a standardized extraction form. The following variables were collected from each included study: author, year, country, study design, target population, setting, intervention or integration model, mental health and HIV components, reported outcomes, facilitators, and barriers.

For consistency, the term “adolescents and young people (AYP)” was applied to participants aged 10–24 years, even when studies used alternative terms such as “youth.” Integration approaches were grouped into three broad categories (fully integrated, partially integrated, and referral-based) to simplify comparison across diverse contexts.

	Article 1	Article 2	Article 3	Article 4
**Citation (Title, Author, Year)**				
**Objective**				
**Methodology/Design**				
**Target population**				
**Location/Setting**				
**Intervention/Integration model/s**				
**Key findings, including reported outcomes, barriers, and facilitators**				
